# Numerical Analysis of Flexural Behavior of Prestressed Steel-Concrete Continuous Composite Beams Based on BP Neural Network

**DOI:** 10.1155/2022/5501610

**Published:** 2022-05-10

**Authors:** Huanhuan Du, Jianyou Pan, Huaxun Shen, Jie Dong

**Affiliations:** ^1^Wenzhou Key Laboratory of Intelligent Lifeline Protection and Emergency Technology for Resilient City, College of Architecture and Energy Engineering, Wenzhou University of Technology, Wenzhou 325035, China; ^2^Eye Hospital, Wenzhou Medical University, Wenzhou 325035, China; ^3^Zhejiang Daily Digital Culture Technology Development Co., Ltd, Hangzhou 310039, China; ^4^Daishan County Bureau of Housing and Urban-rural Development, Zhoushan 316000, China

## Abstract

Prestressed steel-concrete continuous composite beam (PCCB) is a kind of beam, which makes reinforced concrete slab and steel beam bear load and coordinate deformation through connectors such as studs. Prestressed steel-concrete continuous composite beam is a kind of transverse load-bearing composite member formed by prestressed technology on the basis of ordinary composite beam. Aiming at the flexural behavior of prestressed steel-concrete continuous composite beams, a three-dimensional finite element numerical analysis model is established, and the whole process of the test is simulated based on BP neural network. The calculated results are in good agreement with the test. Using this model, the mechanical deformation performance of prestressed steel-concrete continuous composite beam is further analyzed, and the effects of some parameters (steel beam strength grade, concrete strength grade, concrete slab thickness, and transverse reinforcement ratio) on the flexural performance of prestressed steel-concrete continuous composite beam are discussed, which provides a reference basis for engineering design.

## 1. Introduction

Prestressed steel-concrete continuous composite beam is a kind of transverse load-bearing composite member based on ordinary composite beam and prestressed technology. Prestressed steel-concrete continuous composite beam has the advantages of ordinary composite beam. At the same time, it increases the elastic working range of the composite beam, improves the ultimate bearing capacity of the structure, improves the fatigue and fracture performance, increases the strength reserve, improves the reliability of the beam, delays the concrete cracking, and prolongs the service life of the structure. Since the late 1980s, scholars from various countries have begun to study prestressed continuous composite beams. In 1990, Troitsky studied the theory and design of prestressed steel bridges [1]. In 2005, Dall Asta and Zona studied the external prestressed composite beam and deformation connector, established a nonlinear finite element model according to the test, and compared the calculation results with the test results [2]. Other researchers conducted theoretical and experimental research on this structural form for many times [3–5]. In 2021, Zhou and Wang [6] and others studied the influence of prestress on the stress distribution characteristics of flexural section of composite beam.

At present, the research of prestressed steel-concrete continuous composite beams is mostly limited to the negative moment region, and there is still a lack of systematic test and theoretical research for the whole beam. Due to the complexity of mechanical properties of prestressed steel-concrete continuous composite beams, most of the existing calculation models are mainly self-programmed, and the accuracy and stability of calculation have not been fully verified by experiments. In view of this, in order to reveal the stress and deformation mechanism of prestressed steel-concrete continuous composite beam, a three-dimensional nonlinear finite element numerical analysis model of prestressed steel-concrete continuous composite beam considering the longitudinal slip between the interface between concrete and steel beam is established by using the finite element software ANSYS. The whole process of the test is numerically simulated, and the finite element calculation results are compared with the test results. The rationality of the numerical analysis model is verified. Using this model, the mechanical performance and failure mechanism of prestressed steel-concrete continuous composite beams are analyzed and studied. The effects of some parameters (steel beam strength grade, concrete strength grade, steel beam web thickness, loading mode, concrete slab thickness, and longitudinal and transverse reinforcement ratio) on the mechanical performance of prestressed steel-concrete continuous composite beams are discussed, which provides a reference for engineering design.

The rest of the paper is structured as follows. In Section 2, we analyze the research status of prestressed steel-concrete continuous composite beams in recent years. In Section 3, the finite element model of prestressed steel-concrete continuous composite beam is analyzed. In Section 4, the finite element simulation of prestressed steel-concrete continuous composite beam is carried out. In Section 5, the experimental simulation of the proposed method is carried out. The concluding remarks are presented in Section 6.

## 2. Related Works

In the research of prestressed steel-concrete composite beams, Lou and Karavasilis evaluated the flexural performance of two-span prestressed steel-concrete composite beams and quantified the secondary bending moment. The nonlinear behavior of prestressed composite beam is simulated and verified by a continuous test [7]. Chen conducted experimental research on four groups of prestressed steel-concrete composite beams with external tendons in the negative moment area and conducted experimental research on the cracking performance and ultimate negative moment resistance of composite beams [8]. Chen tested four full-scale continuous composite beams (two-span beams and three-span beams) [9]. Lou et al. considered the change of tendon force and adopted the limit equilibrium method to establish the equilibrium equation, avoiding the cumbersome simultaneous solution of deformation coordination equation [10]. Wang et al. proposed a new type of continuous composite beam composed of steel box girder and ultra-high performance concrete waffle grating. Ultra-high performance concrete helps to improve the ultimate bearing capacity and span of the structure and reduce the risk of cracks in ordinary concrete [11].

Neural network has strong parallelism and adaptability and can be applied to control, information, prediction, and so on. Chaudhary et al. proposed a method to predict the inelastic moment of continuous composite beams from elastic moment using neural network. The proposed neural network model predicts the inelastic moment ratio at the span support [12]. Kumar et al. proposed a closed form expression to quickly predict the long-term deflection of simply supported steel-concrete composite bridges under service load. The expression considers the flexibility, shear lag effect, and time effect of shear connectors in concrete [13]. Jasim and Al-Jabbri discussed the application of artificial neural network in the design of continuous composite beams by the plastic method [14]. Pendharkar et al. used the neural network model to quickly predict the inelastic moment in the high-rise composite frame [15]. Kalibhat and Upadhyay proposed two different cross section improvers and the proposed simplified numerical program that has advantages in flexibility, versatility, and ease of use [16]. Kim et al. proposed a simplified method to easily use the effect of thermal prestress method in design, so as to reduce the computational workload of fine analysis [17].

In view of this, aiming at the flexural performance of prestressed steel-concrete continuous composite beams, a three-dimensional finite element numerical analysis model is established, and the whole test process is simulated based on BP neural network. At the same time, using this model, the mechanical deformation performance of prestressed steel-concrete continuous composite beam is further analyzed, and the effects of some parameters on the flexural performance of prestressed steel-concrete continuous composite beam are discussed, which provides a reference basis for engineering design.

## 3. Finite Element Model of Prestressed Steel-Concrete Continuous Composite Beam

### 3.1. Experimental Description

**Figure 1:**
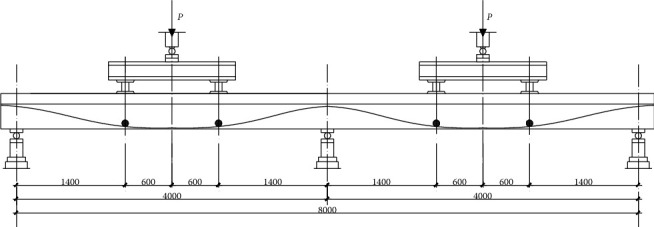
Loading diagram.

**Figure 2:**
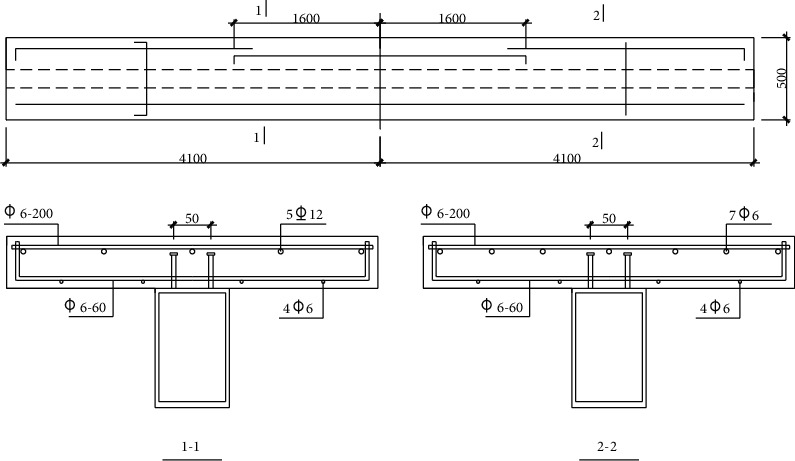
Reinforcement of concrete slab.

In this paper, four specimens are selected as the research objects. The specimens are four-point bending prestressed steel-concrete continuous composite beams with studs as complete shear connectors. The structure and basic parameters of the test piece are shown in Figures 1 and 2 and Tables 1 and 2, respectively.

There are two types of reinforcement in concrete slab: HRB335 reinforcement with a diameter of 12 mm and a yield strength of 368mpa; HPB235 grade reinforcement has a diameter of 6 mm and has been cold drawn, with a yield strength of 372 MPa. The spacing between the stud ends of the other members is 361.80 mm, and the ultimate strength of the stud within the shear stud section is 361.80 mm. The area of single prestressed reinforcement is 139 mm^2^ and the ultimate stress is 1860 MPa.

### 3.2. Three-Dimensional Finite Element Model

**Figure 3:**
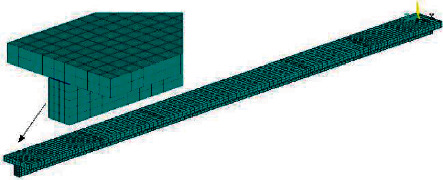
Finite element model diagram.

According to the test process, this paper establishes the ANSYS three-dimensional finite element numerical analysis model of prestressed steel-concrete continuous composite beam, as shown in Figure 3. The concrete slab and steel beam are connected by studs. Therefore, when dividing the grid, it is necessary to ensure that there are corresponding nodes between the concrete slab and steel beam at the location of studs, so as to generate stud elements. In the model, the concrete adopts three-dimensional 8-node solid element SOLID65, which can consider the cracking and crushing of concrete at the same time. The steel beam adopts three-dimensional four-node plate shell element shell43. The bolt connection adopts the nonlinear spring element combin39, which is arranged along the *Z* direction. The combin39 element is set to translate along the *Z* axis and couple the degrees of freedom in the *X* and Y directions. Therefore, each node can slip and deform along the *Z* axis under the constraint of the bolt. The displacement constraints in *X*, *Y*, and *Z* directions are imposed on the joint at the support in the composite beam. At the joint at the end support of the composite beam, the displacement constraints in *X* and *Y* directions are applied. The external load is equivalent to the node load and applied at the corresponding position in the composite beam span.

The ideal elastoplastic model (BISO) and von Mises yield criterion are adopted for steel beams, reinforcement, and concrete. The elastic modulus is 2 × 10^5^ MPa. Poisson's ratio is 0.3, and the yield strength is selected according to the measured value.

Concrete is defined by the nonlinear elastic material model (MISO), and the failure criterion is Willam–Warnke five-parameter failure criterion. In the characteristic parameter table of concrete element, the crack opening shear transfer coefficient is 0.5 and the crack closing shear transfer coefficient is 0.95 [18]. The constitutive relation of concrete under uniaxial stress adopts the expression suggested by the literature [19]. Cracking stress *f*_*t*_ is 0.26*f*_cu_^2/3^.

The uniaxial stress-strain relationship of prestressed reinforcement is calculated by

(1) $$\begin{array}{c} \sigma =\frac{\varepsilon }{E_{0}}+0.002{\left(\frac{\varepsilon }{f_{0.2}}\right)}^{13.5}, \end{array}$$

where *E*_0_=1.95 × 10^5^, *f*_0.2_=0.85*f*_*b*_, and *f*_*b*_ is the ultimate stress of prestressed reinforcement. The initial yield point of prestressed reinforcement is taken as the proportional limit (*f*_*e*_=0.75*f*_*b*_).

The shear slip curve of the stud shows obvious nonlinear characteristics. This paper adopts the stud model proposed in the literature [20, 21]:

(2) $$\begin{array}{c} V=V_{u}{\left(1-e^{ns}\right)}^{m}, \end{array}$$

where *V* is shear force, *s* is slip, *m* = 0.558, *n* = 1 mm^−1^, and *V*_*u*_ is the ultimate bearing capacity of a single stud, which can be calculated by [22]

(3) $$\begin{array}{c} V_{u}=0.43A_{us}\sqrt{E_{c}f_{c}}\leq 0.7A_{us}f_{u}, \end{array}$$

where *f*_*u*_ is the ultimate tensile strength of the stud, *A*_*us*_ is the cross-sectional area of the stud, and *E*_c_ is the elastic modulus of the concrete. *f*_*c*_=0.76*f*_*u*_.

On the basis of considering the cracking and crushing of concrete, the plastic deformation of steel beam and the slip effect between concrete and steel beam, the Newton Raphson equilibrium iterative method, is adopted in this paper. In this method, the load substep is set to 400 steps, and the force load and displacement convergence criteria are adopted.

## 4. Finite Element Numerical Analysis of Prestressed Steel-Concrete Continuous Composite Beam Based on BP Neural Network

### 4.1. Establishment of BP Neural Network Model

**Figure 4:**
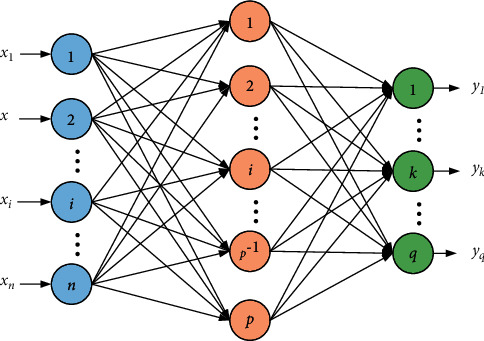
BP neural network infrastructure.

The BP algorithm of back error propagation is called BP algorithm [23, 24]. Its learning process consists of two aspects: error back feedback and information forward transmission. The input layer information is transferred from the input layer to the hidden layer and then processed and transferred to the output layer, that is, each layer of neurons is the input of the next layer of neurons. When there is a large error between the output result of the output layer and the expected value, the error information is fed back in reverse, and the threshold and weight are modified and connected through the neurons of each layer according to the feedback result, so as to achieve the purpose that the error value decreases along the gradient direction and finally achieve the purpose that the error between the output value and the expected value meets the requirements. The BP neural network infrastructure is shown in Figure 4.

Suppose there are *P* training samples in total, and the network trains the *N*th sample pair. The input value is *x*_*i*_, the hidden value is *y*_*j*_, and the output value is *O*_*k*_. The weights and thresholds between the hidden layer and the input layer are *w*_*ji*_ and *θ*_*j*_, respectively. The weights and thresholds between the output layer and the hidden layer are *w*_*kj*_ and *θ*_*k*_, respectively. The expected value of the output layer is *t*_*k*_. For the *N*th sample, the error value between the network output value *O*_*k*_ and the expected value *t*_*k*_ is represented by *E*.

The output of the hidden node is expressed as

(4) $$\begin{array}{c} y_{i}=f\left(\sum_{i}w_{ji}\cdot x_{i}-\theta_{j}\right)=f\left({\text{net}}_{j}\right). \end{array}$$

The calculated output of the output node is expressed as

(5) $$\begin{array}{c} O_{k}=f\left(\sum_{j}w_{kj}\cdot y_{j}-\theta_{k}\right)=f\left({\text{net}}_{k}\right). \end{array}$$

The error formula of the output node is expressed as

(6) $$\begin{array}{c} E=\frac{1}{2}\sum_{k}{\left(t_{k}-O_{k}\right)}^{2}. \end{array}$$

The BP neural network design training calculation process is mainly composed of the following steps:

1. Select the number of hidden layers and their neurons and establish a neural network model.
2. The error function used in training and the conditions for determining termination are given.
3. Input training samples to train the network in step (1).
4. Compare the output value with the real value and adjust the weight and threshold.
5. Bring the results obtained in step (4) into the calculation. If the conditions given in step (2) are met, use the test data group and verification data group for verification. If not, repeat steps (2)–(4) or change the network model.
6. Finally, the trained network is output.

### 4.2. Finite Element Numerical Simulation of Composite Beam

**Figure 5:**
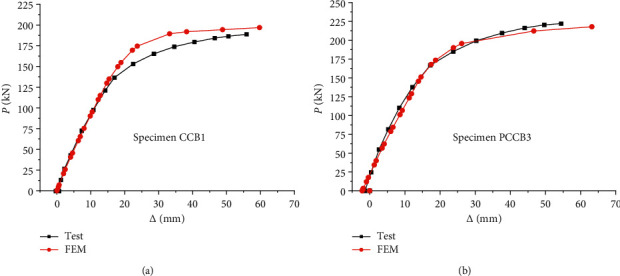
Comparison between calculated curve (*P* − Δ) value and test value.

Based on the neural network algorithm described in the previous section, we use the three-dimensional finite element numerical analysis model established in this paper to carry out nonlinear numerical simulation of the whole test process and calculate the load midspan deflection curve (*P* − Δ), as shown in Figure 5 and Table 3. In the figure, *P* is the load value and Δ is the midspan deflection value. It can be seen from the figure that the development trend of the curve obtained by finite element calculation is roughly the same as that of the test curve, which is in good agreement.

It can be seen from Figure 4 that the calculated results are in good agreement with the test results. Therefore, the three-dimensional finite element numerical analysis model of prestressed steel-concrete continuous composite beam established in this paper is reasonable. Prestressed tendons are mainly used as tensile members in the work of composite beams, which give full play to the tensile performance of reinforcement. The prestressed reinforcement of beam body is generally divided into three types: longitudinal prestressed reinforcement, vertical prestressed reinforcement, and transverse prestressed reinforcement. In this paper, longitudinal prestressed reinforcement refers to the longitudinal pressure applied to the lower part of the beam. This is to reduce the tensile stress at the lower part of the beam, improve the bearing capacity of the composite beam, and expand its elastic working range under the action of vertical load. The calculation results show that the bearing capacity of prestressed continuous composite beams is increased by 10.7% compared with ordinary continuous composite beams.

Taking PCCB3 specimen as an example, this paper explains the stress mechanism of prestressed steel-concrete continuous composite beam. Before the load is applied to the composite beam, the prestressed tensioning is carried out first, and the reverse arch appears in the middle of the span during the tensioning process. Prestressed steel-concrete continuous composite beam is a kind of statically indeterminate structure. Its midspan internal force and support reaction are determined by the stiffness distribution of the composite beam. When the bending moment in the negative bending moment area of the middle support exceeds the pressure dissipation bending moment established in advance before loading, the concrete on the upper part of the middle support cracks, the stiffness decreases, and the internal force redistributes. The nonlinear finite element method can accurately simulate the whole stress process of composite beam, which is mainly divided into the following four stages:

1. Prestressing and structural reverse arch stage: for ordinary steel-concrete continuous composite beams, the test of composite beams directly starts from monotonic loading to failure of composite beams. For the prestressed continuous composite beam, the prestressed tension shall be carried out before loading (the support shall be centered and tightened with the loading device in the test). The loading can be carried out only after the prestressed reinforcement is tensioned to the control value and the anchorage is completed until the composite beam is damaged.
2. Elastic working stage: at the initial stage of loading, the midspan curve *P* − Δ basically develops in a linear relationship. At this stage, the prestressed continuous composite beam is in the elastic stage and has good working performance. At this stage, the nonlinear finite element calculation curve of prestressed steel-concrete continuous composite beam fits well with the test curve. Comparing the two specimens, it can be seen that the yield load of prestressed composite beam is about 0.87 *P*_*u*_, which is greater than the end point of elastic section of ordinary steel composite beam by 0.65 *P*_*u*_.
3. Elastoplastic working stage: with the continuous increase of load, the deflection growth rate of prestressed steel-concrete continuous composite beam is accelerating, and the curve *P* − Δ presents a typical nonlinear law. At this time, prestressed steel-concrete continuous composite beam has entered the elastic-plastic working stage.
4. Failure stage: when the load is close to the ultimate load, the prestressed continuous composite beam shows that the tension of the midspan section of the steel beam reaches the yield stage, while the compression of the midspan concrete slab reaches the ultimate load and the concrete is crushed. Plastic hinges appear in many places of prestressed continuous composite beams, resulting in a sharp decline in the bearing capacity of composite beams. At this time, the composite beam is in the plastic working stage.

**Figure 6:**
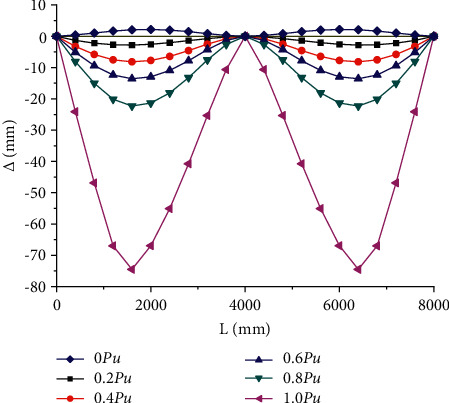
Distribution curve of deflection of PCCB3 specimen along beam length.

Figure 6 shows the distribution of deflection of composite beam along the beam length of PCCB3 specimen in different stress stages. It can be seen from the figure that the maximum deflection appears on the left of the midspan. The deformation development process can be divided into three stages: elastic deformation stage (*P* ≤ 0.6 pu), elastic-plastic deformation stage, and plastic deformation stage. The maximum slip value caused by prestressed inverted arch occurs at the sliding support, and the maximum value is −0.76 mm. The maximum slip value in the elastic stress stage is 0.97 mm, the maximum slip value in the elastic-plastic stress stage is 1.64 mm, and the maximum slip value in the plastic stress stage is 5.09 mm. The development range of slip value in plastic stress stage accounts for 67.78% of the total slip value.

**Figure 7:**
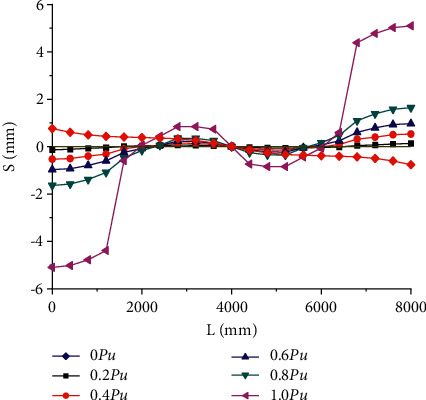
Distribution curve of PCCB3 specimen slip along beam length.

Figure 7 shows the distribution of longitudinal horizontal slip along the beam length of the composite beam interface at different stress stages of PCCB3 specimen. The specimen beam is a composite beam with complete shear connection. It is found that the horizontal slip curve has an antisymmetric distribution centered on the middle support, and the slip value is approximately zero. At the same time, the slope of the slip curve in the midspan area is large, the slip mutation is fast, and the slip change at both ends is gentle. From the analysis of loading process, at the initial stage of loading, the slip value is small and develops slowly. With the increase of load, the slip value increases continuously. When the bending moment exceeds 0.6 pu, the development rate of horizontal slip is significantly accelerated. In the limit state, the maximum horizontal slip value of the interface is 5.09 mm. The maximum slip value caused by prestressed inverted arch occurs in the middle of the span, and the maximum value is 2.16 mm. The maximum deflection value in the elastic stress stage is −13.57 mm, the maximum deflection value in the elastic-plastic stress stage is −22.31 mm, and the maximum deflection value in the plastic stress stage is −74.50 mm. It can be seen from the data that the deflection value produced in the final plastic deformation stage changes obviously, and it shows that prestress is helpful to prolong the elastic working range of composite beams.

## 5. Experimental Analysis

**Figure 8:**
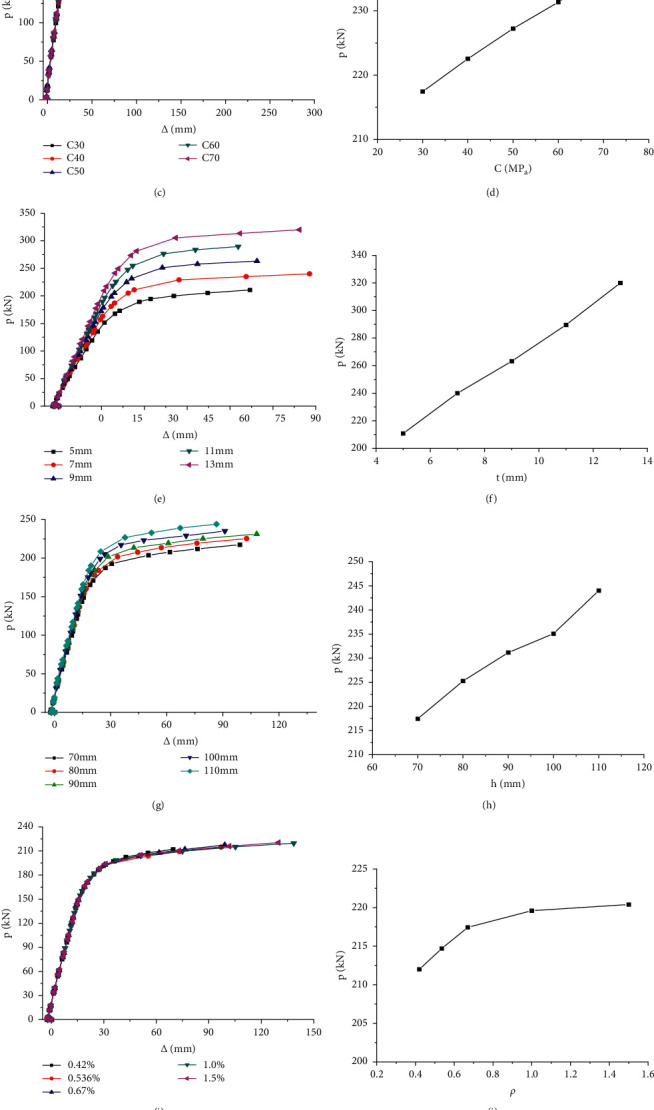
Influence of parameter changes on *P* − Δ curve. (a) Specimens with different steel beam strength grade. (b) *P* changes with steel beam grade. (c) Specimens with different concrete strength grade. (d) *P* changes with concrete grade C. (e) Specimens with different web thickness of steel beam. (f) *P* changes on the web thickness of steel beam. (g) Specimens with different thickness variation of concrete slab. (h) *P* changes on the concrete slab thickness h. (i) Specimens with different transverse reinforcement ratio. (j) *P* changes longitudinal reinforcement ratio. (k) Specimens with different loading mode.

Taking PCCB3 beam as the reference beam, this paper discusses the effects of steel beam strength grade, concrete strength grade, web thickness, concrete slab thickness, and longitudinal and transverse reinforcement ratio on the flexural performance of prestressed steel-concrete continuous composite beam, as shown in Figure 8.

The strength grade of steel beam has obvious influence on the flexural capacity of prestressed steel-concrete continuous composite beam, as shown in Figure 8(a). When the strength of the steel beam is increased from 235 MPa to 420 MPa, its flexural bearing capacity is 210.8 kNm, 255 kNm, 282.4 kNm, and 304 kNm, respectively. Therefore, using high-strength steel, the flexural capacity of composite beams has been effectively improved, and the increase is obvious.

With the increase of concrete strength grade, the flexural capacity of composite beam has been improved to a certain extent, but the increase range is slow. When the concrete strength is increased from C30 to C40, the flexural capacity of composite beam is improved relatively greatly, and when the concrete grade is increased from C40 to C70, the bearing capacity is improved slightly. At the same time, the increase of concrete strength has little effect on the stiffness of composite beam in elastic working stage. In addition, the ductility of composite beam also increases with the increase of concrete strength grade, as shown in Figure 8(b) Therefore, concrete with appropriate strength grade should be selected in engineering design. In this paper, C30 ∼ C50 grade concrete is recommended.

With the increase of the web thickness of the steel beam, the flexural capacity and stiffness of the composite beam increase, especially for the stiffness in the elastic working stage, as shown in Figure 8(c). Therefore, in engineering design, selecting thicker steel beam web is a way to effectively improve the flexural capacity of composite beams.

With the increase of concrete slab thickness, the flexural capacity and stiffness of composite beams increase. The influence mechanism is to increase the height of the compression zone of the concrete slab to improve the flexural capacity of the composite beam, as shown in Figure 8(d). Therefore, selecting thicker concrete slab in engineering design is a way to effectively improve the flexural capacity of composite beams.

Increasing the transverse reinforcement ratio of concrete slab can improve the flexural capacity of composite beams to a certain extent, but the effect is not obvious. With the increase of transverse reinforcement ratio of concrete slab, the ductility of specimens is continuously improved. It shows that the increase of stirrup reinforcement ratio can not only restrict the deformation of concrete but also better promote the cooperative work of concrete and steel beam, as shown in Figure 8(e).

When using different loading methods in the calculation of composite beams, it can be seen that the effect of composite beams on different loading methods is very different. Through the comparison of the two curves in Figure 8(f), it can be seen that the stiffness and bearing capacity of the composite beam under the uniformly distributed load are far greater than those under the concentrated load. Therefore, the composite beam is designed according to the stress characteristics of components in practical engineering.

## 6. Conclusion

In this paper, aiming at the mechanical performance of prestressed steel-concrete continuous composite beam, the whole process is simulated by numerical analysis method, and the failure mechanism, bearing capacity, and main influencing factors of prestressed steel-concrete continuous composite beam are analyzed. The main results are summarized as follows:

1. High-strength steel is used in composite beams, which has high flexural capacity and superior plastic deformation capacity.
2. Increasing the concrete strength grade can increase the flexural capacity and ductility of composite beams, but the influence degree decreases with the further increase of concrete strength. Considering certain economic performance, this paper suggests to select C30 ∼ C50 strength concrete in engineering design.
3. Increasing the thickness of concrete slab and steel beam web can effectively improve the flexural capacity of composite beams. Among them, the increase of web thickness of steel beam is more obvious to improve the stiffness of composite beam in the elastic working stage.
4. Increasing the transverse reinforcement ratio can improve the ultimate bearing capacity of composite beams. The increase of stirrup reinforcement ratio can not only restrict the deformation of concrete but also better promote the cooperative work of concrete and steel beam.
5. Under different loading modes, the action effects of composite beams are obviously different. When designing the composite beam, it is based on the actual stress characteristics of the composite beam.

At present, the mechanical performance, dynamic performance, fatigue performance, construction performance, and seismic performance of prestressed rigid concrete continuous composite beams under long-term effect are rarely involved in the current research. The research team will carry out the above related experimental research in the next step.

## Figures and Tables

**Table 1:** Measured tensile strength of steel.

Steel plate thickness (mm)	5	6	8
Yield strength of lower flange of steel beam (*f*_*y*_/MPa)	287.7	249.3	272.3

**Table 2:** Parameters of the specimens.

Specimen no.	Prestressed tendon no.	Arrangement of prestressed reinforcement	Compressive strength of concrete (*f*_*y*_/MPa)	Total prestress (To/kN)
CCB1	0	None	34.1	0
PCCB3	1	Broken line layout	31.3	133.75

**Table 3:** Comparison between calculated value and test value of ultimate bearing capacity.

Specimen no.	Test value	Calculated value	Test value/calculated value
CCB1	188.7	196.804	1.04
PCCB3	222.5	217.83	0.98

## Data Availability

The data used to support the findings of this study are available from the corresponding author upon request.
